# Monte Carlo Study of the abBA Experiment: Detector Response and Physics Analysis

**DOI:** 10.6028/jres.110.067

**Published:** 2005-08-01

**Authors:** E. Frlež

**Affiliations:** abBA Collaboration, Department of Physics, University of Virginia, Charlottesville, VA 22904-4714, USA

**Keywords:** detector Monte Carlo simulation, neutron beta decay, GEANT4

## Abstract

The abBA collaboration proposes to conduct a comprehensive program of precise measurements of neutron *β*-decay coefficients *a* (the correlation between the neutrino momentum and the decay electron momentum), *b* (the electron energy spectral distortion term), *A* (the correlation between the neutron spin and the decay electron momentum), and *B* (the correlation between the neutron spin and the decay neutrino momentum) at a cold neutron beam facility. We have used a GEANT4-based code to simulate the propagation of decay electrons and protons in the electromagnetic spectrometer and study the energy and timing response of a pair of Silicon detectors. We used these results to examine systematic effects and find the uncertainties with which the physics parameters *a*, *b*, *A*, and *B* can be extracted from an over-determined experimental data set.

## 1. Introduction

The abBA collaboration is proposing to perform a measurement of a “complete set” of correlations in the neutron *β*-decay using the same apparatus, and improve the precision of the correlation coefficients *a*, *b*, *A*, and *B* by up to an order of magnitude.

GEANT4 is a general-purpose software package for simulation of the passage of particles through matter that provides a complete set of tools for all domains of detector simulation [1]. In particular, the GEANT4 toolkit currently provides particle tracking in nonuniform magnetic and electric fields and handles combined electromagnetic fields transparently [2]. The GEANT4 Low Energy Electromagnetic Physics group validates the low energy electromagnetic processes for electrons down to 250 eV [3].

In this report we describe a GEANT4 simulation of the abBA spectrometer and outline the algorithm for the extraction of the physics decay parameters.

## 2. abBA Detector Geometry

In the tentative design of the abBA spectrometer the decay particles (electrons and protons) are guided by the electric and magnetic fields and interact only in sensitive detectors thus avoiding the energy losses and scatterings in apertures, grids, or windows [4].

**Fig. 1:**
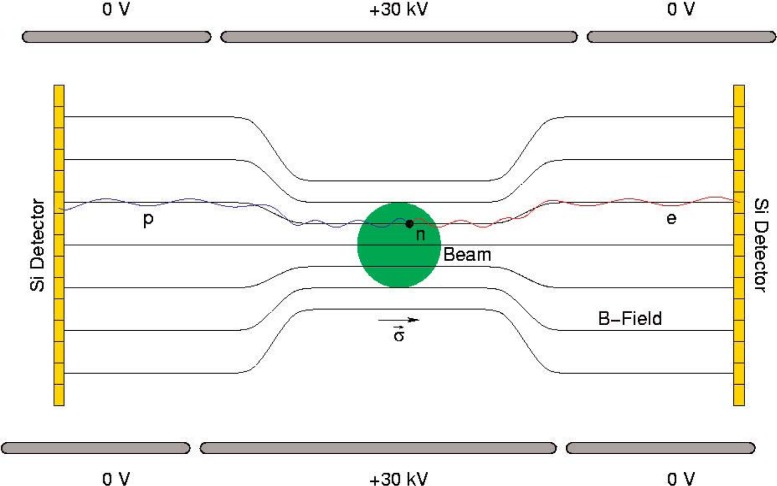
The schematic drawing of the main elements of the abBA spectrometer.

The simplified geometry of the detector defines two sensitive planar Silicon detectors with a 100 mm × 100 mm^2^ area and a 2 mm thickness. The two Si detectors are separated by 4 m. The coordinate system is defined with the Si detectors at *x*_1,2_ = ± 2 m (Fig. 1).

A passive solenoid magnet is placed around the decay region, with its axis of symmetry perpendicular to the incident neutron beam, along the *x* coordinate. The 3 m long magnet with a 0.8 m radius can produce a 4 T central magnetic field that decreases to 1 T at the detector positions, thus guiding charged particles from the decay region to the Si detectors. A tubular electrode held at ≈ 30 kV accelerates the protons so they can be detected in a silicon detector.

## 3. Magnetic Field

The magnetic field along the *x* axis of the detector solenoid is given by:

$$B(x,\rho =0)=\frac{2\pi NI}{c}\left(\frac{L-x}{\sqrt{R^{2}}+{\left(L-x\right)}^{2}}+\frac{x}{\sqrt{R^{2}+x^{2}}}\right),$$

where *L* is the length of the solenoid, *R* its radius, and *x* the axial coordinate. Meanwhile, *N* denotes the number of turns per unit length, and *I* is the electrical current in the closely wound cylindrical coil.

Thanks to axial symmetry, the magnetic field off-axis, outside its sources, can be represented in terms of the magnetic field strength *B*(*x*, 0) along the axis:

$$\begin{array}{l} B_{x}(x,\rho )=\frac{\partial \phi }{\partial x}=\sum_{n=0}^{\infty }\frac{{\left(-1\right)}^{n}}{{\left(n!\right)}^{2}}B^{\left(2n\right)}(x){\left(\frac{\rho }{2}\right)}^{2n}\\ \;=B(x)-\frac{\rho^{2}}{2}B^{\prime \prime }(x,0)+\cdots , \end{array}$$

and

$$\begin{array}{l} B_{\rho }(x,\rho )=\frac{\partial \phi }{\partial \rho }=\sum_{n=1}^{\infty }\frac{{\left(-1\right)}^{n}}{(n-1)!n!}B^{\left(2n-1\right)}(x){\left(\frac{\rho }{2}\right)}^{2n-1}\\ \;=\frac{\rho }{2}B^{\prime }(x,0)+\cdots , \end{array}$$

where *ϕ* is the magnetic scalar potential and $\rho =\sqrt{y^{2}+z^{2}}$ is the axial radius coordinate. These fields have been programmed into the GEANT4 user routine.

## 4. Event Generator

The electrons from the neutron *β*-decay are generated from (5 × 5 × 5) mm^3^ central volume with the relativistic differential decay rate given by [5]:

$$\frac{d\Gamma }{dE_{e}d\Omega_{p_{e}}d\Omega_{p_{\nu }}}=\frac{{\left(G_{F}V_{\text{ud}}\right)}^{2}}{{\left(2\pi \right)}^{5}}\frac{F(E_{e})|p_{e}|E_{\nu }}{m_{n}[E_{p}+E_{\nu }+E_{e}(\beta \cdot {\hat{p}}_{\nu })]}{\left|M\right|}^{2},$$

where *E*_e_ and ***p***_e_ (*E*_ν_ and ***p***_ν_) are the electron (neutrino) energy and momentum, *m*_n_ is the neutron mass, *G*_F_ is the Fermi constant, *V*_ud_ is the Cabbibo-Kobayashi-Maskawa matrix element, ***β*** = ***p***_e_/*E*_e_, and *F*(*E*_e_) is the Fermi function that describes the interaction of the electron and the recoil proton.

The transition matrix element squared |*M*|^2^ is given by

$$\begin{array}{l} {\left|M\right|}^{2}=m_{n}m_{p}E_{e}E_{\nu }\left(1+\frac{\alpha }{2\pi }e_{V}^{R}\right)\left(1+\frac{\alpha }{2\pi }\delta_{\alpha }^{\left(1\right)}\right)\\ \;\times C_{0}(E_{e})\left(1+3{\tilde{g}}_{A}^{2}\right)\{1+\left(1+\frac{\alpha }{2\pi }\delta_{\alpha }^{\left(2\right)}\right)C_{1}\left(E_{e}\right)\beta \cdot {\hat{p}}_{\nu }\\ \;+b\left(\frac{m_{e}}{E_{e}}\right)+\left(1+\frac{\alpha }{2\pi }\delta_{\alpha }^{\left(2\right)}\right)[C_{2}(E_{e})+C_{3}\left(E_{e}\right)\beta \cdot {\hat{p}}_{\nu }]\hat{n}\cdot \beta \\ \;+[C_{4}(E_{e})+C_{5}(E_{e})\beta \cdot {\hat{p}}_{\nu }]n\cdot p_{\nu }\} \end{array}$$

where *m*_p_ is the proton mass, *α* is the fine structure constant, $e_{V}^{R}$ is a low energy constant, *δ_α_*’s are model-independent radiative corrections, ${\tilde{g}}_{A}$ is the axial coupling constant, and the correlation coefficients *a*, *A*, *B* are incorporated into the recoil corrections *C_i_*(*E*_e_) [6].

## 5. Results and Conclusions

A GEANT4 simulation of abBA detector energy and timing response was performed for 10^6^ neutron *β*-decays. We used the values of the correlation coefficients from Ref. [7] (*a* = −0.1039, *b* = 0, *A* = −0.1161, *B* = 0.9878). For each event we recorded the neutron polarization, generated momenta of the final state particles and measured energy depositions and timing hits in the Silicon detectors.

**Fig. 2:**
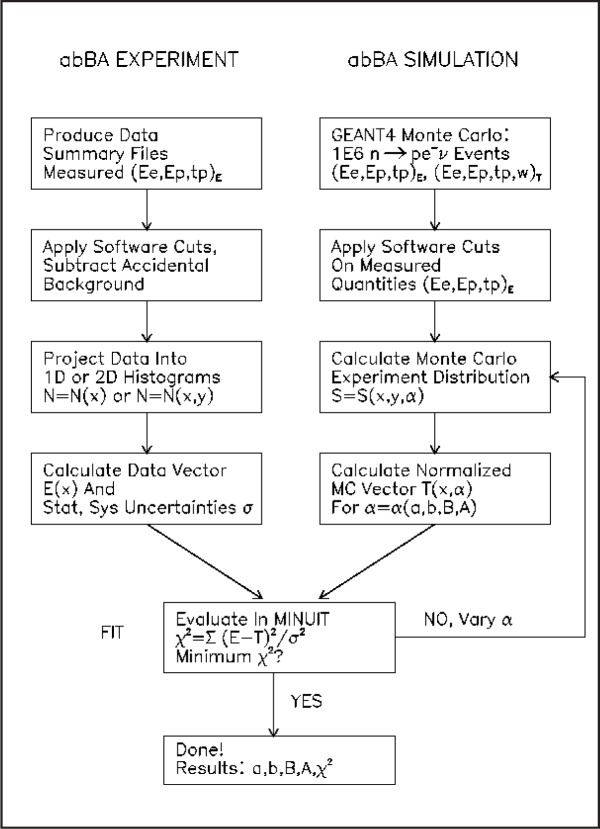
A flow diagram of the analysis. Monte Carlo histograms are recalculated in each step of MINUIT minimization. The “experiment” depends only on measured quantities (subscript *E*), while the MC column depends both on generated variables (subscript *T*) and simulated detector response (subscript *E*).

The separate GEANT4 run which included systematic effects (energy and timing resolutions of Si detectors, detector calibration uncertainties, detector response nonlinearities, magnetic field inhomogeneities, neutron polarization uncertainty, etc.) was used to simulate the experimental data. (“MC data”). The flow chart of the physics analysis is summarized in Fig. 2. The correlation coefficients and their fitted uncertainties are extracted using the standard MINUIT code [8].

**Fig. 3:**
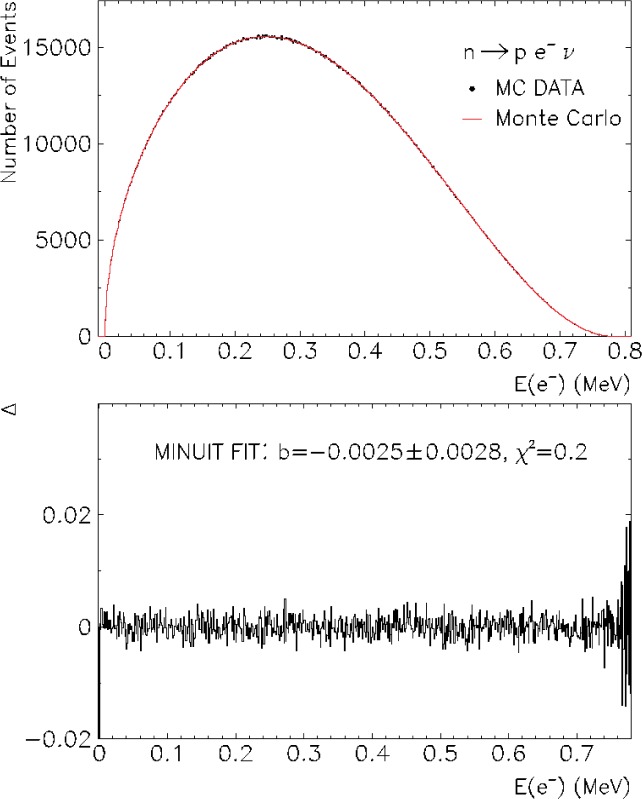
Monte Carlo energy spectrum of neutron *β*-decay electron (top) and fractional differences *Δ* = (*exp* − *the*)/(*exp* + *the*) between the simulated and “MC data” spectrum (bottom).

**Fig. 4:**
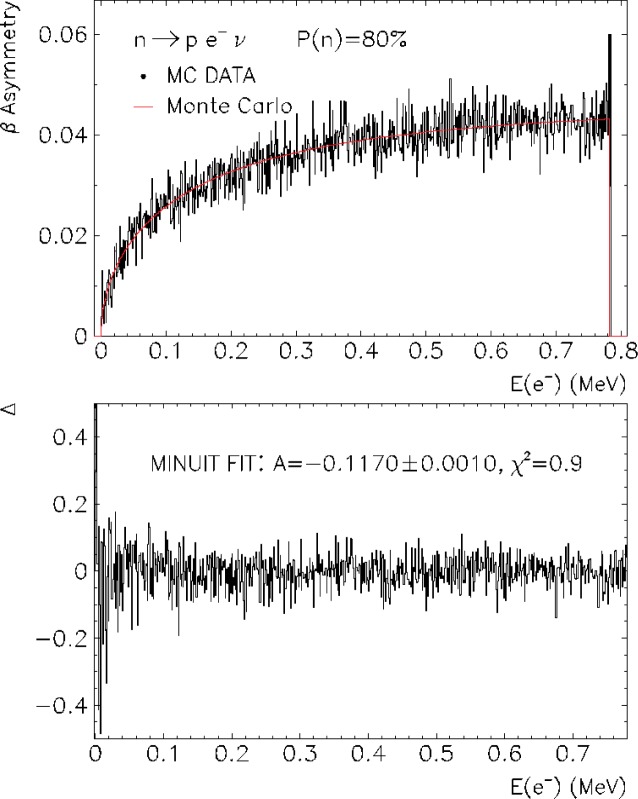
Monte Carlo histogram of the *β* asymmetry (top) and fractional differences between the simulated and “MC data” spectrum as a function of the electron energy (bottom).

We present two example results: (i) the extraction of the parameter *b* with unpolarized neutron beam in Fig. 3, and (ii) the asymmetry coefficient *A* for 80 % polarized neutron beam in Fig. 4. At the current stage of development, a GEANT4 simulation limited to 10^6^ neutron decay events and 10^6^ MC data events, runs 24 CPU hours on a 1 GHz Linux computer. Given the limited event statistics, analysis of MC data results in the coefficient *b* = −0.0025 ± 0.0028 and the coefficient *A* = −0.1170 ± 0.0010, where statistical and systematic uncertainties are combined. The code will be made faster by using the adiabatic invariants for charged particle tracking in the electromagnetic field, which will markedly improve the uncertainties of our method.
